# Regulation of transcriptome plasticity by mTOR signaling pathway

**DOI:** 10.1038/s12276-025-01508-y

**Published:** 2025-08-14

**Authors:** Jeongsik Yong, Hyunjoo Kim, Euyeon Lee, Yoonju Jung

**Affiliations:** 1 Department of Biochemistry, Molecular Biology and Biophysics, University of Minnesota Twin Cities, Minneapolis, MN USA; 2 Department of Chemical Engineering and Material Sciences, University of Minnesota Twin Cities, Minneapolis, MN USA; 3 Department of Medicine, School of Medicine, Chosun University, Gwangju, Republic of Korea

**Keywords:** Molecular biology, Alternative splicing

## Abstract

The mechanistic target of rapamycin (mTOR) pathway, long recognized for its critical roles in cellular metabolism and growth, is increasingly appreciated for its regulatory impact on the transcriptome. Recent insights into mTOR’s regulation of alternative splicing and polyadenylation reveal a sophisticated mechanism by which mTOR influences RNA processing to affect the proteome’s diversity and functionality. Here, in this Review, we delve into the multifaceted roles of mTOR in modulating transcriptome plasticity, highlighting its influence beyond traditional functions such as protein synthesis and cell growth. By examining the latest findings, we explore how mTOR-mediated transcriptome plasticity plays a pivotal role in cellular adaptation and pathogenesis. Studies indicate that mTOR modulation of RNA processing pathways enables cells to respond dynamically to environmental and metabolic cues, thereby altering protein function and cellular behavior in a context-dependent manner. This capability is crucial for both normal physiological responses and the development of disease. The Review also discusses the implications of these findings for understanding complex biological systems and diseases, particularly cancer, where mTOR’s regulation of transcript diversity could drive tumor heterogeneity and treatment resistance. As research continues to uncover the extensive influence of mTOR on RNA processing, it becomes clear that a comprehensive understanding of these mechanisms is essential for the development of targeted therapies and the prediction of their outcomes in clinical settings.

## mTOR pathway shapes RNA processing and protein diversity

The mechanistic target of rapamycin (mTOR) pathway is crucial for controlling various cellular processes, including how genes are expressed. This Review explores how mTOR affects RNA processing, specifically alternative splicing and polyadenylation. The authors review how mTOR influences these processes and their impact on protein production. The study highlights that mTOR operates through two complexes, mTORC1 and mTORC2, which have different roles in cell growth and survival. mTORC1 is sensitive to nutrients and promotes protein synthesis, while mTORC2 supports cell survival. The research shows that mTOR affects gene expression by altering RNA processing, which can lead to changes in protein function. The findings suggest that the regulation of RNA processing by mTOR is crucial for understanding diseases such as cancer and metabolic disorders. Future research could explore how targeting mTOR-related pathways might offer new treatments for these conditions.

This summary was initially drafted using artificial intelligence, then revised and fact-checked by the author.

## Introduction

Cellular signaling pathways orchestrate a multitude of biological processes. Among such pathways, the mechanistic target of rapamycin (mTOR) pathway plays a pivotal role in regulating transcriptome plasticity, including phenomena such as alternative splicing and alternative polyadenylation (APA). In this Review, we delve into the intricate mechanisms by which mTOR influences these aspects of RNA processing and examine the subsequent functional consequences on the proteome. We also explore emerging insights into transcriptome plasticity that extend beyond the field’s traditional boundaries, offering a fresh perspective on how gene expression is modulated at a posttranscriptional level. Through this exploration, we aim to illuminate the broader implications of mTOR signaling in cellular function and disease and provide a comprehensive overview of current knowledge and future directions in studying transcriptome plasticity.

## Mammalian target of rapamycin pathway and its relevance in human diseases

### mTOR complexes

**Fig. 1:**
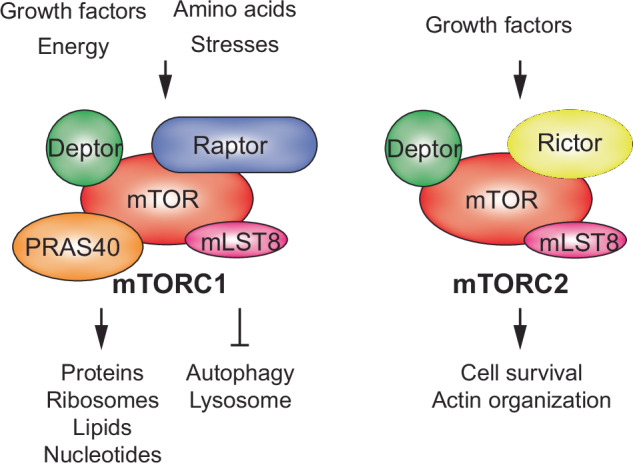
mTOR complexes, along with their key upstream regulators and downstream target pathways. The diagram illustrates representative components of mTORC1 and mTORC2.

mTOR is a serine/threonine protein kinase in the PI3K-related kinase (PIKK) family, vital for cellular signaling and energy metabolism^1^. mTOR operates through two distinct multiprotein complexes, mTOR complex 1 (mTORC1) and mTOR complex 2 (mTORC2), which differ in component composition, substrate specificity and sensitivity to rapamycin, a well-known mTOR inhibitor^2^. mTORC1, consisting of mTOR, regulatory-associated protein of mTOR (Raptor), and mammalian lethal with Sec13 protein 8 (mLST8), is acutely sensitive to rapamycin^2^ (Fig. 1). This complex primarily promotes cell growth and proliferation by facilitating anabolic processes, including protein synthesis, lipid synthesis and ribosome biogenesis, and by inhibiting catabolic processes such as autophagy^1^ (Fig. 1). The activation of mTORC1 depends heavily on nutrient availability, growth factors and cellular energy levels, positioning it as a critical regulator of cellular metabolism^3^. mTORC2, meanwhile, includes mTOR, the rapamycin-insensitive companion of mTOR (Rictor) and mLST8^4^ (Fig. 1). Unlike mTORC1, mTORC2 is mainly resistant to rapamycin and influences cell survival, cell cycle progression and cytoskeletal organization. It plays a crucial role in activating AKT, a key player in cell survival pathways, and supports the integrity of the actin cytoskeleton^5^ (Fig. 1).

Both complexes are implicated in various human diseases; mTORC1 has been linked to cancers, obesity, diabetes and neurodegenerative diseases owing to its role in promoting cell growth and inhibiting autophagy. mTORC2 has been implicated in cancers and insulin resistance, highlighting its role in cell survival and metabolism^6^.

### Upstream regulators and downstream target pathways of mTOR

The mTOR pathway is tightly regulated by various upstream signals that communicate cellular and environmental conditions. These regulators ensure that mTOR activity reflects the needs and status of the cell, coordinating growth and metabolic responses appropriately. mTORC1 activity is susceptible to the presence of amino acids, which activate it via Rag–Ragulator complex-mediated translocation to lysosomal membranes, a key site for mTORC1 activation^7^. This localization is essential for mTORC1 to effectively integrate signals from nutrient status to modulate growth and metabolism^7^.

Insulin and growth factors stimulate the PI3K–Akt signaling pathway, a primary upstream regulator of mTORC1 and mTORC2^1^. The activation of PI3K leads to the production of PIP3, which recruits AKT to the membrane, where it can be activated by phosphorylation. Active AKT directly phosphorylates and inhibits TSC2, a negative regulator of mTORC1, thus promoting mTORC1 activity^8^. mTORC2 is also involved in the full activation of AKT by phosphorylating it at Ser473, further enhancing its downstream signaling capabilities^5^. Cellular energy levels, indicated by AMP/ATP ratios, are sensed by AMPK, which inhibits mTORC1 activity through phosphorylation of TSC2 and Raptor, demonstrating the critical role of mTOR in linking energy status with growth regulation^9^.

mTORC1 is a critical regulator of protein synthesis, a fundamental process underpinning cellular growth and proliferation^10^ (Fig. 1). This regulation is achieved predominantly through phosphorylating key targets involved in the initiation and elongation phases of mRNA translation, specifically 4E-BP1 and S6K1^11^. The modulation of these targets by mTORC1 not only enhances general protein synthesis but also selectively increases the translation of specific classes of mRNAs crucial for ribosome biogenesis and cellular metabolism^11^.

One of the primary mechanisms by which mTORC1 enhances protein synthesis is the phosphorylation of 4E-binding proteins (4E-BPs). Under conditions of low mTORC1 activity, 4E-BP1 is in a hypophosphorylated state and binds strongly to eIF4E, a cap-binding protein essential for initiating cap-dependent translation. This binding inhibits the assembly of the eIF4F complex, composed of eIF4E, eIF4A (an ATP-dependent RNA helicase) and eIF4G (a scaffold protein), thereby reducing protein synthesis. When mTORC1 is activated by nutrients or growth factors, it phosphorylates 4E-BP1, releasing it from eIF4E. The liberated eIF4E can then bind to the 5′ cap structure of mRNA, facilitating the recruitment of other components of the eIF4F complex^12^. This assembly is crucial for the unwinding of mRNA secondary structures in the 5′ untranslated regions (UTRs), a prerequisite for the scanning process of the 40S ribosomal subunit and the subsequent initiation of translation^12^.

mTORC1 activity specifically enhances the translation of mRNAs that contain 5′-terminal oligopyrimidine tracts (5′-TOP) and polypyrimidine-rich translational elements (5′-PRTE)^13^. These elements are typically found in the leaders of mRNAs encoding ribosomal proteins and components of the translation apparatus, making their translation sensitive to changes in mTORC1 activity. The phosphorylation of 4E-BP1 and the consequent activation of cap-dependent translation are crucial for the efficient translation of these mRNAs. This selective translation is vital for expanding the cellular protein synthesis machinery, especially under conditions favoring growth^13^.

In addition to regulating eIF4E, mTORC1 also activates S6 kinase 1 (S6K1). Upon activation, S6K1 phosphorylates several substrates, including the ribosomal protein S6 (rpS6), which enhances the translation of mRNAs with oligopyrimidine tracts in their 5′-UTRs^14^. Furthermore, S6K1 phosphorylates and activates eIF4B, which assists eIF4A in unwinding RNA structures during the initiation phase. The activation of eIF4B by S6K1 thus enhances the helicase activity of eIF4A, promoting the translation of mRNAs with highly structured 5′-UTRs^15^.

The dual actions of mTORC1 on 4E-BP1 and S6K1 integrate to promote protein synthesis robustly. This integrated control ensures that cells can rapidly and effectively increase their protein synthesis capacity in response to growth signals, thereby supporting cellular proliferation and growth. This regulation by mTORC1 is essential for normal cellular functions and implicates its dysregulation in various diseases, including cancer, where unchecked cellular growth and protein synthesis drive tumor progression.

In lipid metabolism, mTORC1 and mTORC2 play distinct roles. mTORC1 regulates adipogenesis and lipid synthesis via direct phosphorylation of targets such as Lipin1, while mTORC2 is crucial for the systemic regulation of glucose and lipid homeostasis^16,17^. These processes are vital in maintaining cellular energy balance and are often dysregulated in metabolic diseases such as diabetes and obesity^18^. The role of mTOR in angiogenesis is mediated through different mechanisms in mTORC1 and mTORC2. mTORC1 promotes angiogenesis through the stabilization of HIF-1α and the activation of VEGF, critical in conditions like rheumatoid arthritis where new blood vessel formation is enhanced^19^. Conversely, mTORC2 contributes to angiogenesis by activating AKT and eNOS, promoting endothelial cell function and vascular health^20^. Autophagy, a key catabolic process involving the degradation and recycling of cellular components, is intricately regulated by mTORC1^21^. In nutrient-rich conditions, mTORC1 inhibits autophagy through phosphorylation of ULK1 and other elements critical for autophagosome formation^22^. Conversely, nutrient deprivation or cellular stress inhibits mTORC1, promoting autophagy and maintaining cellular homeostasis^22^.

### Role of mTOR in human pathogenesis

mTOR signaling plays a pivotal role in the development and progression of cancer. The PI3K–AKT–mTOR pathway is frequently activated in cancer owing to mutations in upstream regulators such as PTEN, PI3K or AKT^23^. This activation leads to increased cell proliferation, growth and survival. In addition, mTORC2 contributes to oncogenic processes by reorganizing the cytoskeleton and enhancing cell migration and invasion, facilitating tumor metastasis^24^. In metabolic disorders such as diabetes and obesity, mTOR signaling is often hyperactivated, contributing to pathogenesis through mechanisms such as insulin resistance^25^. mTORC1 activation leads to the phosphorylation of S6K1, which can promote insulin resistance by phosphorylating and degrading IRS-1, a key insulin signaling component. This pathway’s dysregulation disrupts normal glucose and lipid metabolism, exacerbating the metabolic syndrome^26^. mTOR signaling is crucial in neurological disorders owing to its role in protein synthesis, neuronal growth and autophagy. Overactivation of mTOR has been linked to the accumulation of pathogenic proteins, such as β-amyloid in Alzheimer’s disease and α-synuclein in Parkinson’s disease^27^. In addition, disruptions in mTOR signaling are implicated in psychiatric disorders such as schizophrenia and autism spectrum disorders, where they may affect neurogenesis and synaptic plasticity^28^. Inflammation involves the innate immune response, where mTOR can regulate the function of mast cells, phagocytes and natural killer cells^29^. The pathway modulates the production of inflammatory cytokines through factors such as STAT3 and NFkB, suggesting that mTOR inhibitors could serve as potential anti-inflammatory agents^30^. The role of mTOR in human pathogenesis extends beyond the pathways discussed above. Given its involvement in various biological processes, the importance of mTOR in various pathological conditions is likely to expand as ongoing research uncovers new functions.

## Transcriptome plasticity through the regulation of alternative splicing and APA

This section delves into the intricate world of posttranscriptional regulation of gene expression, focusing on the pivotal roles of alternative splicing and APA. These processes are central to the diversification of the transcriptome and, consequently, the proteome, enabling cells to adapt to various physiological demands and environmental changes. By altering mRNA splicing patterns and poly(A) tailing locations, cells can finely tune gene expression in a context-dependent manner, highlighting the complexity and versatility of genetic regulation.

### Splicing and alternative splicing during pre-mRNA processing

Pre-mRNA splicing is a crucial gene expression process in the cell nucleus, where introns (noncoding regions) are removed and exons (coding regions) are joined to form mRNA. This mRNA then serves as a template for protein synthesis^31^. This sets the stage for alternative splicing, a complex posttranscriptional mechanism that allows a single gene to produce multiple protein isoforms by selectively including or excluding certain exons and introns. This modification in exon assembly enhances proteome complexity and genetic diversity without increasing the gene count. Crucial for development, function and adaptation in organisms, alternative splicing influences various biological processes and plays a significant role in disease pathogenesis.

**Fig. 2:**
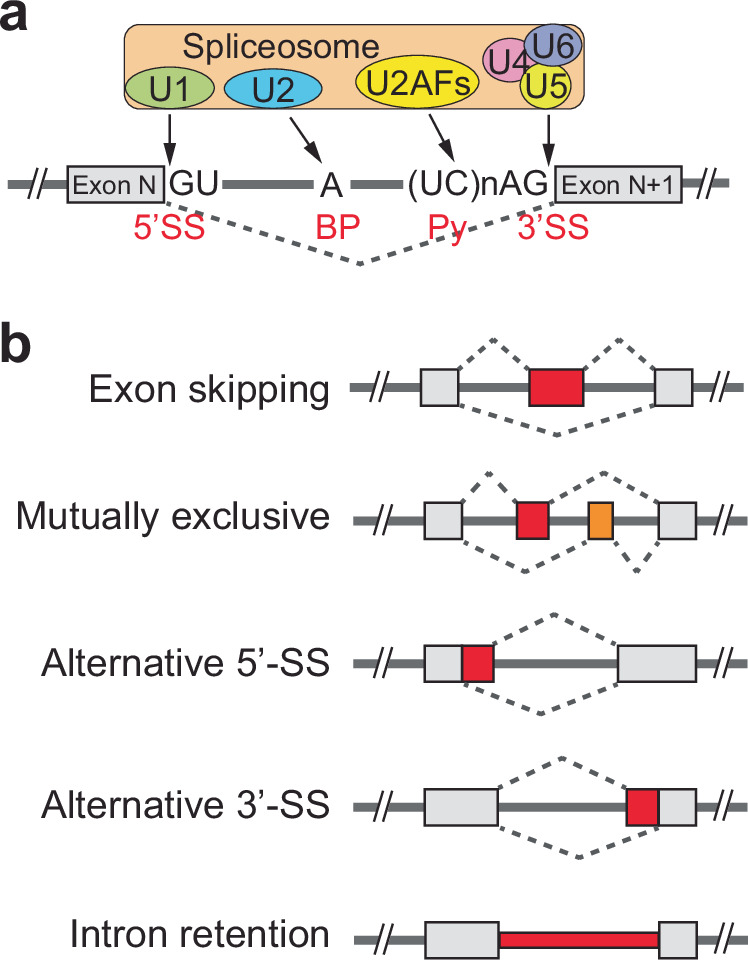
Schematic of splicing and alternative splicing. **a** An illustration of *cis*- and *trans*-acting factors involved in pre-mRNA splicing, highlighting four essential *cis*-acting elements recognized by U snRNPs and splicing factors, including U2AF heterodimers. **b** Classification of alternative splicing types, with exon skipping also referred to as cassette-type splicing.

The core *cis*-acting elements crucial for splicing include the 5′-splice sites (5′SS), the branch point (BP), polypyrimidine tracts (Py) and the 3′-splice sites (3′SS). The 5′SS, located at the start of an intron, features a conserved GU sequence in eukaryotes, vital for initiating splicing^32^. This site is recognized by specific spliceosomal components that facilitate the correct folding and alignment of the RNA for accurate intron removal and exon joining. The branch point, typically an adenosine nucleotide situated 20–50 nucleotides before the 3′SS, is pivotal for the splicing reaction. It anchors the formation of a lariat structure that is integral to the splicing mechanism, serving as the attack point for the first nucleophilic reaction that removes the intron. The polypyrimidine tract, following the branch point and leading up to a conserved AG sequence at the intron’s end, aids in the recognition and proper alignment of the spliceosome to the 3′SS, ensuring the intron is precisely cut and the exons are smoothly connected^32^ (Fig. 2a).

Spliceosome, a complex molecular assembly composed of small nuclear ribonucleoproteins (snRNPs) and numerous other proteins, dynamically recognizes and binds these *cis*-elements to execute splicing. This molecular machine undergoes a series of assembly, rearrangement and catalytic steps involving extensive RNA–RNA, RNA–protein and protein–protein interactions^33^. Key spliceosomal snRNPs—U1, U2, U4, U5 and U6—play distinct roles: U1 snRNP binds to the 5′SS, while U2 snRNP attaches to the branch point, crucial for the integration and activation of the spliceosome (Fig. 2a). The coordination among these snRNPs, along with numerous other splicing factors such as serine/arginine-rich (SR) proteins and heterogeneous nuclear ribonucleoproteins (hnRNPs), regulates the assembly of the spliceosome, splicing accuracy and the selection of alternative splice sites^33^. These factors adjust the splicing machinery in response to cellular cues and developmental signals, contributing to the diversity of mRNA and protein products.

Alternative splicing is a versatile mechanism by which a single pre-mRNA transcript is processed into multiple isoforms, significantly expanding the proteomic complexity and functional capacity of an organism. Exon skipping, the most common form of alternative splicing, involves the omission of an exon, leading to mRNA variants with or without specific protein-coding sequences, which impacts the protein’s structure and function. Intron retention, another type, occurs when one or more introns are not excised from the mRNA, potentially introducing premature stop codons or altering the reading frame, which can result in nonfunctional proteins or regulatory RNAs. Mutually exclusive exons involve choosing one exon over an adjacent alternative in the final mRNA, each variant imparting different functional properties to the proteins. Alternative splicing can also occur at the 5′SS or 3′SS, where using different donor or acceptor sites results in mRNAs with varied 5′ or 3′ ends, affecting both the mRNA’s coding potential and regulatory elements^32^ (Fig. 2b).

The regulation of alternative splicing involves a complex interplay of factors. Splicing factors such as SR proteins and hnRNPs play key roles in these decisions. They either promote or inhibit the spliceosome assembly at specific splice sites by binding to regulatory sequences within the pre-mRNA^34^. Intronic and exonic splicing enhancers and silencers influence splice site selection by either attracting or repelling splicing factors^35^. Moreover, external signals from cellular signaling pathways can modify the activity of splicing factors through changes in their phosphorylation state, thereby impacting splicing outcomes^36,37^. The secondary structure of RNA itself can influence splicing by making certain sites more or less accessible to the splicing machinery, and RNA-binding proteins (RBPs) can bind specific RNA motifs to affect splicing patterns, often integrating various cellular signals^38^.

The intricate relationship between alternative splicing and disease pathology is a critical area of research within molecular biology and genetics. By modulating splicing events, alternative splicing factors can significantly influence the onset, progression and severity of various diseases by altering gene expression patterns and protein functions. In oncology, misregulation of alternative splicing is increasingly acknowledged as a key factor in tumor development and malignancy. Splicing factors such as SF3B1, SRSF1 and U2AF1 are frequently mutated in various cancers, leading to aberrant splicing patterns that produce oncogenic isoforms^39^. These changes in splicing can result in the skipping of tumor suppressor exons or the inclusion of oncogenic exons, thereby enabling unchecked cellular proliferation, evasion of apoptosis and increased metastatic potential^39,40^. Alternative splicing also plays a significant role in the central nervous system, particularly concerning several neurodegenerative disorders. Mutations that affect splicing regulators or splice site recognition can produce dysfunctional proteins critical for neuronal survival and function. For example, in spinal muscular atrophy and amyotrophic lateral sclerosis, alterations in the splicing of genes such as *SMN2* result in decreased levels of essential proteins, undermining motor neuron maintenance and function^41^. The impact of alternative splicing extends to cardiovascular health, where it is involved in crucial processes such as cardiac muscle development and response to myocardial stress. Genetic variants that affect the splicing of transcripts coding for proteins such as titin can lead to cardiomyopathies and heart failure^42^. These splicing abnormalities can alter the mechanical properties of heart muscle, resulting in impaired cardiac function. With profound implications, aberrant splicing can modify immune responses in inflammatory and autoimmune diseases^43,44^.

### Polyadenylation and APA during pre-mRNA processing

**Fig. 3:**
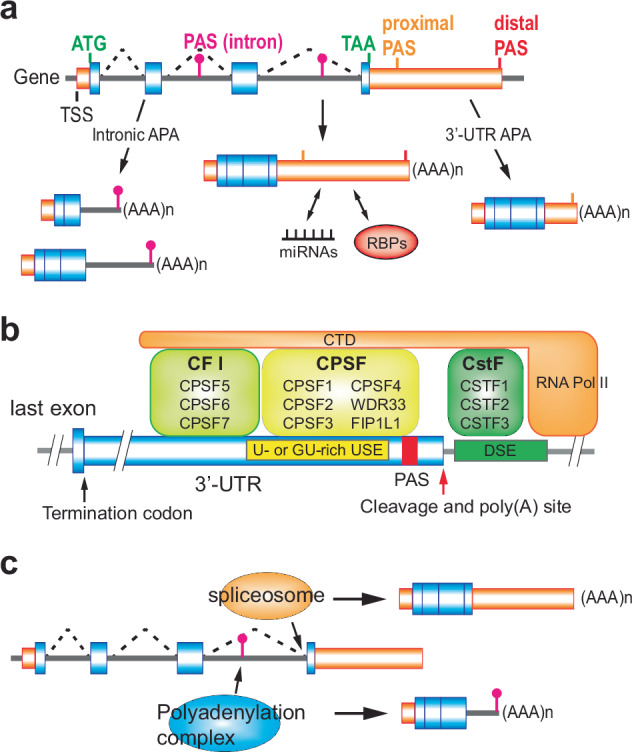
Schematic of APA. **a** There are two main types of APA. The first type, known as 3′-UTR APA, occurs within the 3′-UTR of a gene and does not alter the gene’s coding capacity but may result in shortened transcripts that evade regulation by miRNAs and RBPs. The second type occurs within introns, leading to truncated mRNAs and potentially truncated proteins. **b** *Cis*- and *trans*-acting factors in polyadenylation: multiple polyadenylation factors recognize distinct sequence elements around the polyadenylation signal (PAS) in 3′-UTRs. **c** Intronic polyadenylation utilizes PASs found in introns. The choice between splicing and intronic polyadenylation is influenced by the cellular and disease context, with both processes competing for the same pre-mRNA.

Polyadenylation is a critical posttranscriptional process that involves the addition of a poly(A) tail to the 3′ end of an RNA molecule. This modification occurs co-transcriptionally, and variations in the polyadenylation site and poly(A) tail length are critical for RNA stability, nuclear export and translational efficiency^45^. The poly(A) tail also plays a crucial role in regulating the lifespan of the mRNA within the cytoplasm. APA is a variation of this process that can produce mRNA molecules of different lengths from the same gene using different polyadenylation sites^45^. This leads to the generation of mRNA isoforms with varying 3′-UTRs (3′-UTR APA) and, occasionally, upstream intron regions (intronic APA)^46^ (Fig. 3a).

Polyadenylation of the pre-mRNA process is governed by specific sequence elements within the mRNA (*cis*-acting elements) and various proteins (*trans*-acting factors) that recognize and interact with these sequences to execute polyadenylation. Polyadenylation occurs at the 3′ end of pre-mRNA molecules and begins with identifying a polyadenylation signal, most commonly the hexanucleotide sequence AAUAAA, located upstream of the cleavage site. This signal is crucial as it directs the assembly of the polyadenylation machinery at the correct site on the pre-mRNA. Following this signal sequence, a less conserved downstream sequence element (DSE), typically U-rich or GU-rich, helps define the cleavage site for polyadenylation. The assembly of the polyadenylation machinery involves several key *trans*-acting factors. The cleavage and polyadenylation specificity factor (CPSF) binds to the AAUAAA signal and is essential for recognizing and cleaving the pre-mRNA^47^. The cleavage stimulation factor (CstF) attaches to the DSE, facilitating the interaction between CPSF and the pre-mRNA^48^ (Fig. 3b). Together, these complexes coordinate the cleavage of the pre-mRNA at a site typically 10–30 nucleotides downstream of the polyadenylation signal^49^. After cleavage, poly(A) polymerase adds a tail of adenine residues to the newly created 3′ end. This poly(A) tail can vary in length but generally consists of about 200 adenosine residues in eukaryotic cells. The addition of the poly(A) tail is critical for enhancing mRNA stability, aiding in nuclear export and facilitating translation initiation. Poly(A) binding protein binds to this tail, further enhancing mRNA stability and translational efficiency^50^.

3′-UTR APA involves using different polyadenylation sites within the 3′-UTR of a gene, leading to the production of mRNA isoforms with varying 3′-UTR lengths. This variation impacts mRNA stability, localization and translational efficiency, thus affecting gene expression levels and the cell’s response to environmental cues^51^. The selection of the polyadenylation site in the 3′-UTR is dictated by the presence and activity of specific polyadenylation factors and by sequence elements within the 3′-UTR, including polyadenylation signals and U-rich and GU-rich elements, which interact with the cellular machinery to determine the precise cleavage and polyadenylation site^51^ (Fig. 3b).

Intronic APA occurs when polyadenylation signals within an intron are utilized, producing truncated mRNA isoforms^46^ (Fig. 3a). This process is intimately linked with splicing, as the machinery for both functions competes for similar signals within the pre-mRNA. Truncated mRNAs are produced if the polyadenylation signal within an intron is recognized and acted upon before the splicing machinery can remove the intron (Fig. 3c). Such premature polyadenylation can lead to nonfunctional mRNAs or those with altered coding capacities, potentially impacting protein function and contributing to disease^46^.

The competition between splicing and polyadenylation is a key regulatory aspect of gene expression. Proteins in both pathways interact with similar *cis*-elements, such as the polypyrimidine tract and specific splicing enhancers or silencers. For instance, CPSFs and CstFs recognize polyadenylation signals that may also affect splice site selection by altering the accessibility or configuration of the pre-mRNA^52^. In addition, splicing factors such as U2AF2 and hnRNPs can influence splice site selection and polyadenylation^52^.

APA significantly impacts human pathogenesis by influencing mRNA stability, localization and translation, thereby affecting the expression and function of crucial genes. This process, which includes altering the length of mRNA 3′-UTRs and generating truncated mRNA from intronic regions, has profound implications across various diseases. In cancer, the shortening of 3′-UTRs by APA in oncogenes such as MYC and BCL2 results in the loss of microRNA (miRNA) binding sites, facilitating the overexpression of these oncogenes and contributing to tumor progression and resistance to apoptosis^53,54^. Similarly, in metastatic cancers, the deregulation of APA can enhance the cells’ ability to metastasize by altering the expression of adhesion molecules and matrix-modifying enzymes^55,56^.

Neurodegenerative disorders such as Alzheimer’s disease and Parkinson’s disease are also affected by changes in APA. For instance, in Alzheimer’s disease, the polyadenylation of transcripts encoding proteins such as APP and BACE1 can alter their expression levels, influencing amyloid plaque formation and disease progression^57^. In Parkinson’s disease, alterations in the polyadenylation of synuclein mRNA can disrupt protein homeostasis, contributing to the characteristic protein aggregation and neuronal death^58^. In cardiovascular diseases, APA affects the expression of genes involved in cardiac muscle function and response to myocardial stress, such as those encoding ion channels and contractile proteins^59^. Aberrant polyadenylation patterns can lead to heart conditions such as cardiomyopathies and heart failure by altering the expression and function of these critical proteins^59^.

3′-UTR APA predominantly disrupts the regulation by *trans*-acting factors binding to 3′-UTR regions, thereby influencing the protein expression levels from different mRNA isoforms. Conversely, intron APA contributes to pathogenesis by producing truncated proteins that may exhibit loss or gain of function, as observed in certain genetic disorders. For example, intron APA within the dystrophin gene can result in shorter, dysfunctional proteins associated with certain forms of muscular dystrophy^60^.

## mTOR-coordinated transcriptome plasticity and its relevance in pathogenic mechanisms

**Fig. 4:**
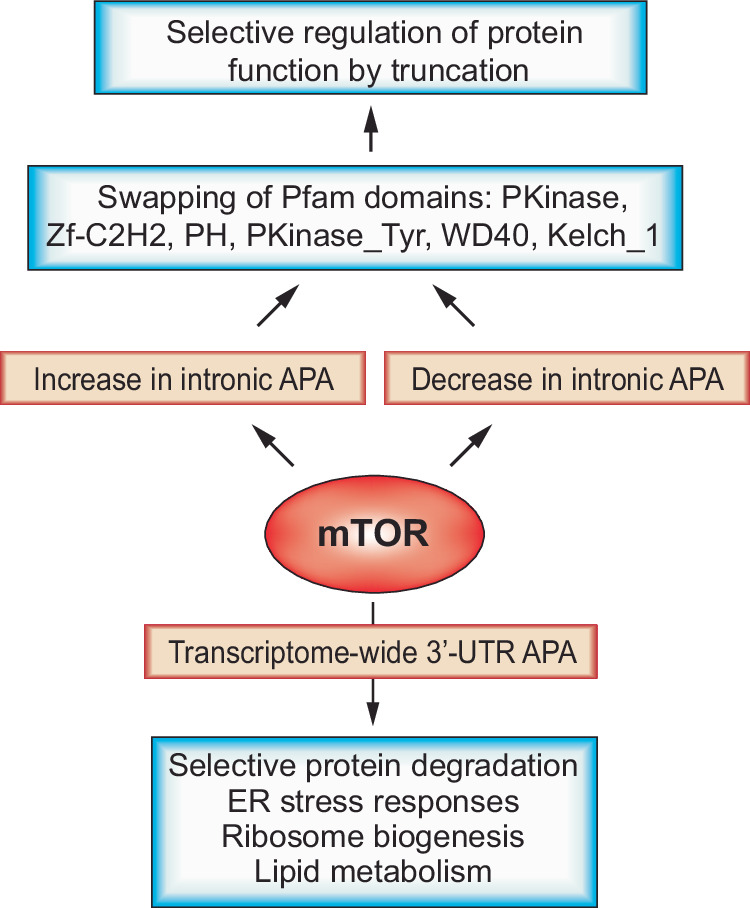
mTOR-regulated APA and its impact on the proteome. mTOR influences the profiles of both 3′-UTR APA and intronic APA. 3′-UTR APA typically enhances translation, whereas intronic APA can lead to both increases and decreases in transcript levels. In cases of intronic APA, the effects on the proteome are predominantly associated with the gain or loss of functional protein domains, significantly altering protein functionality.

Research into mTOR’s role in gene regulation has highlighted its crucial influence on transcriptome plasticity and its potential impact on pathological mechanisms. Studies have shown that mTOR upregulation triggers APA in 3′-UTRs, enhancing protein synthesis. Notably, the discovery of mTOR-regulated pathways influenced by 3′-UTR APA-driven gene expression, such as ubiquitin-mediated proteolysis and the proteasome system, demonstrates that mTOR’s function extends beyond protein synthesis to regulate selective protein degradation^61^ (Fig. 4). Mechanistically, research has demonstrated that mTORC1 controls CPSF6, a crucial polyadenylation factor, by modulating the activities of downstream target kinases. This regulatory mechanism prompts a shift between autophagy and lipid metabolism in cells responding to nutrient stress^62^ (Fig. 4).

Unlike the 3′-UTR APA driven by mTOR, which primarily produces shorter 3′-UTR transcript isoforms, the pattern of intronic APA in the transcriptome varies with changes in cellular mTOR activity. Specifically, when mTOR is activated, some genes exhibit an increase in intronic APA, while others show a decrease^63,64^. This dichotomous pattern emphasizes the intricate role of mTOR in gene expression regulation, illustrating that intronic APA is not just a byproduct of transcription but a tightly regulated process with profound biological and pathological consequences. Evidence of this is seen in the similar patterns of intronic APA observed across various tumor and corresponding normal tissue samples from The Cancer Genome Atlas, which identify genes enriched in intronic APA in a cancer-type-specific manner. Further research on how this APA pattern influences the gain or loss of protein functional domains in pan-cancer The Cancer Genome Atlas data has demonstrated its strategic role in the cancer proteome. This mechanism selectively toggles protein functions by adding or removing domains within the same protein family (Fig. 4). For example, in normal tissues, oncogenic kinases are typically deactivated by removing kinase domains, while anti-oncogenic kinases are activated by adding kinase domains through intronic APA; however, this regulatory pattern is often reversed in tumor samples^64^.

**Fig. 5:**
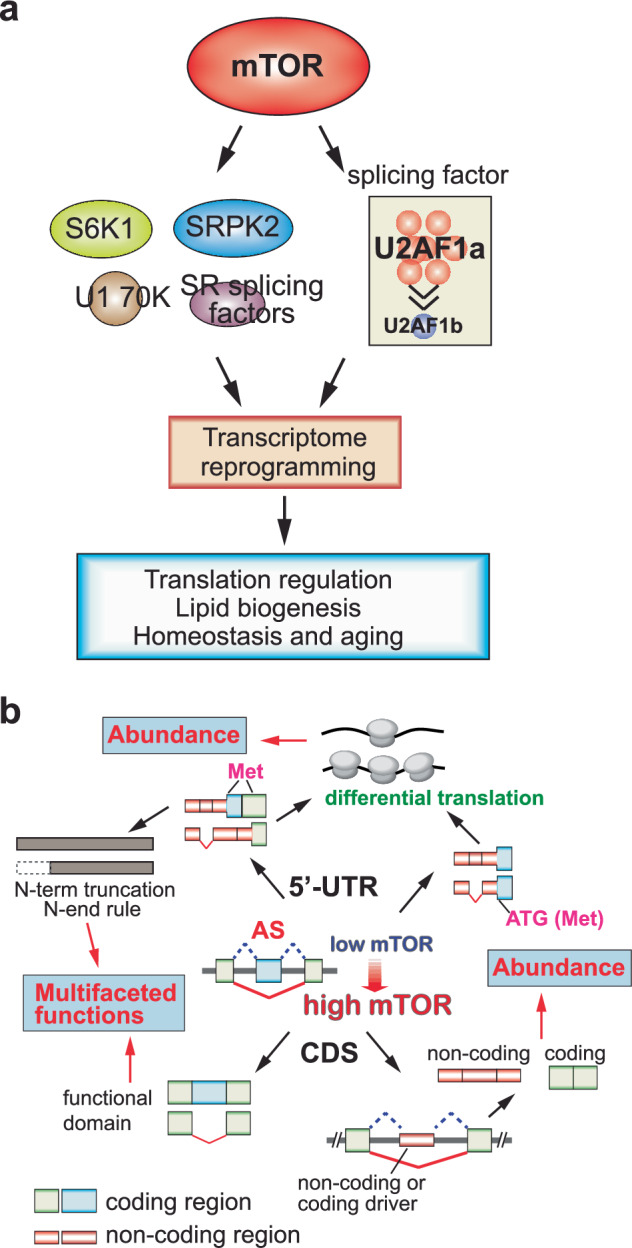
mTOR-coordinated alternative splicing and its impact on the proteome. **a** mTOR influences a variety of splicing factors and RBPs, leading to changes in the alternative splicing patterns of genes involved in lipid biogenesis, cellular homeostasis and lifespan. mTOR also alters the stoichiometry of the essential splicing factor U2AF1, impacting 5′-UTR alternative splicing and modifying protein synthesis efficiency. **b** Generally, mTOR activation promotes exon skipping, which results in varied outcomes for the proteome, dependent on the locations of the alternative splicing events within the mRNAs. 5′-UTR alternative splicing often leads to truncation at the N-terminus of proteins or alters mRNA translational efficiency. In coding regions, alternative splicing can result in the gain or loss of protein functional domains, and sometimes transforms coding mRNA into noncoding transcripts.

mTOR also regulates downstream pathways through the regulation of alternative splicing. By influencing the activity of splicing factors and coordinating cellular responses to nutrient availability, growth signals and stress, mTOR integrates alternative splicing regulation into broader signaling networks. Mechanistically, mTOR’s regulatory effect is highlighted in one of the components of spliceosome machinery, U2 auxiliary factor 1 (U2AF1), which plays a role in selecting 3′SS during pre-mRNA processing. Chang et al. reported that mTOR regulates the expression of U2AF1 isoforms, which display unique molecular profiles in splice site and protein interactions^65^ (Fig. 5a). Their analysis of mutually exclusive alternative splicing events showed that the inherent sequence preferences of U2AF1 isoforms and their relative amounts influence the 3′SS selection. Notably, U2AF1a-led transcriptomes exhibit 5′-UTR alternative splicing events that enhance translation. Their results highlighted the distinct roles of U2AF1 isoforms derived from duplicated tandem exons in transcriptome regulation and indicated that U2AF1a-driven 5′-UTR alternative splicing serves as a mechanism for mTOR-regulated translational control.

Activation of mTOR has been demonstrated to modulate alternative splicing across the transcriptome, typically producing shorter isoform transcripts^66^ (Fig. 5b). This study explored how transcriptome-wide alternative splicing impacts the proteome, finding diverse effects depending on the mRNA regions involved. Alternative splicing at the 5′-UTR or 3′-UTR typically generates proteins with truncated N- or C-termini and affects noncoding region-regulated translation. By contrast, splicing within coding regions often alters the proteome by adding or removing functional domains and regulatory phosphorylation sites in proteins^66^.

A study by Lee et al. demonstrates that mTORC1 facilitates lipid biogenesis through SRPK2, an essential regulator of RNA-binding SR proteins^67^ (Fig. 5a). The activation of S6K1 by mTORC1 leads to the phosphorylation of SRPK2 at Ser494, which is then phosphorylated at Ser497 by CK1. This enhances the nuclear translocation of SRPK2 and its phosphorylation of SR proteins. Inhibition of this signaling pathway results in intron retention in lipogenic genes, ultimately leading to nonsense-mediated mRNA decay. These findings suggest a new role for mTORC1–SRPK2 signaling in the posttranscriptional regulation of lipid metabolism and highlight SRPK2 as a promising therapeutic target for metabolic disorders driven by mTORC1.

Furthermore, a discovery by Huang et al. shows that splicing fidelity profoundly impacts homeostasis and aging^68^ (Fig. 5a). They identified a hypomorphic mutation in the ribonucleoprotein RNP-6, a spliceosomal component essential for recognizing weak 3′SS. This mutation compromises splicing fidelity and specifically leads to intron retention in *egl-8*, which encodes phospholipase Cβ4 (PLCB4), a key regulator of intracellular signaling. The retained intron in *egl-8* reduces its functional transcript and protein levels, thereby dampening PLC signaling. This perturbation activates cellular stress response pathways, which in turn downregulate mTOR signaling—a central regulator of growth and metabolism. Suppression of mTOR activity promotes stress resilience and is well established to enhance longevity. Importantly, they demonstrated that intron retention in *egl-8* is not merely a downstream consequence but a critical effector of lifespan extension in the *rnp-6* mutant background. These findings establish a mechanistic link between splicing fidelity, stress signaling, mTOR modulation and organismal aging^68^.

A recent study by Ogawa et al. showed that mTORC1 kinase plays a crucial role in mRNA splicing and gene expression during larval growth in *C. elegans*^69^. Their genetic and bioinformatic screenings reveal that nutrient availability activates mTORC1, significantly reprogramming gene expression by enhancing the activity of splicing factors such as the SR protein RSP-6 and regulating mRNA splicing independently of its target, p70 S6 kinase. Although their findings provide an exciting insight into mTORC1’s role in regulating alternative splicing, other potentially involved mechanisms should be identified, and further investigation is needed to clarify these mechanisms in humans.

## Conclusions and future challenges: proteogenomic perspectives of mTOR-regulated transcriptome plasticity

mTOR has been a significant focus in biomedical research due to its central role in regulating cell growth, proliferation and survival^5^. Historically, the primary focus on mTOR’s function centered around its role in protein synthesis^10^. In addition to its role in protein synthesis, mTOR has been extensively studied for its involvement in autophagy^21^. Another significant area of focus has been the role of mTOR in aging and cancer. Dysregulation of mTOR signaling is commonly associated with cancer, as it can lead to uncontrolled cell growth and proliferation^70^. Therefore, mTOR inhibitors, such as rapamycin and its analogs, have been widely studied and used in cancer therapy^71^. In addition, research on mTOR’s involvement in lifespan extension, mainly through calorie restriction and reduced protein synthesis, has opened new avenues for studying aging-related diseases and interventions^72^.

Until recently, research on the role of mTOR in gene expression regulation, beyond its well-known effects on protein synthesis, was relatively sparse. It is now understood that changes in mTOR signaling can significantly influence the expression of numerous RBPs, including splicing and polyadenylation factors^66^. This suggests that mTOR plays a crucial role in pre-mRNA processing, contributing to transcriptome plasticity through alternative splicing and polyadenylation. These findings extend the functional scope of mTOR beyond its traditional roles in biology and disease pathogenesis.

Current genome annotations reveal that the human genome comprises fewer than 20,000 genes^73^. This modest number belies the vast functional diversity of the proteome, as well as the specificity and variability of cellular and tissue functions observed during disease progression and across an organism’s lifespan. The flexibility of the transcriptome, driven by mechanisms such as alternative splicing and polyadenylation, allows the production of a multitude of transcript and protein isoforms from a single gene, significantly enhancing the functional capacity of the genome across various biological contexts. Therefore, transcriptome plasticity is essential for maintaining the specificity and multifunctionality of genes.

While numerous studies have examined mTOR’s influence on cellular physiology and disease, the role of alternative isoform production in these processes remains underexplored. Emerging research has begun to highlight mTOR-regulated transcriptome plasticity as a critical contributor to mTOR-associated biology and pathogenesis^74^. Given that nearly the entire transcriptome can exhibit isoform switching under mTOR modulation, there is an urgent need for systematic investigations into the functional importance of specific isoforms in distinct biological contexts.

Specifically, it will be important to identify genes that undergo isoform expression changes in mTOR-driven conditions and to determine how these molecular alterations contribute to physiological outcomes and disease phenotypes. A major challenge will be not only mapping these isoform shifts with high resolution, but also determining their proteogenomic consequences, including changes in protein localization, stability and interactions.

Future studies should aim to develop robust experimental models—such as isoform-specific reporters, knock-in models or CRISPR-based modulation tools—to functionally interrogate these events. In addition, integrating long-read sequencing, single-cell transcriptomics and proteomic data will be essential for accurately capturing the full spectrum of isoform diversity and its dynamic regulation by mTOR. By addressing these gaps, the field stands to uncover a new dimension of mTOR signaling with significant implications for therapeutic targeting and our understanding of cellular adaptability.
